# Long-term outcomes of single-radius total knee arthroplasty in an Asian population: a comparison of cruciate-retaining and posterior-stabilized designs

**DOI:** 10.1186/s43019-026-00349-x

**Published:** 2026-09-04

**Authors:** Gi-Young Jang, Hyobeom Lee, Kang-Il Kim

**Affiliations:** 1 Department of Orthopaedic Surgery, Kyung Hee University Hospital at Gangdong, Seoul, Republic of Korea; 2 Department of Orthopaedic Surgery, School of Medicine, Kyung Hee University, Seoul, Republic of Korea

**Keywords:** Single-radius total knee arthroplasty, Posterior-stabilized, Cruciate-retaining, Long-term follow-up, Survivorship

## Abstract

**Background:**

Single-radius total knee arthroplasty (TKA) has demonstrated favorable clinical outcomes and survivorship; however, long-term comparative data between cruciate-retaining (CR) and posterior-stabilized (PS) designs within a single-radius system remain limited, particularly in Asian populations. This study aimed to evaluate the long-term clinical and radiologic outcomes and survivorship of single-radius TKA and to compare CR and PS designs in an Asian population.

**Methods:**

This retrospective study included 222 knees (152 patients) that underwent primary TKA using a single-radius prosthesis between 2006 and 2015, with a minimum follow-up of 10 years. There were 112 CR knees and 110 PS knees. Clinical outcomes were assessed using the Knee Society Score, Western Ontario and McMaster Universities Osteoarthritis Index, and Forgotten Joint Score. Radiologic outcomes were also evaluated. Kaplan–Meier survivorship analysis was performed using revision for all causes and aseptic revision as endpoints. Inverse probability of treatment weighting (IPTW) was applied to adjust for baseline differences between groups.

**Results:**

At a mean follow-up of 13.2 ± 2.8 years, significant improvements were observed in all clinical outcomes compared with preoperative values (all* p* < 0.001). At the final follow-up, clinical outcomes did not differ significantly between the CR and PS groups, and IPTW-adjusted analyses also showed no statistically significant between-group differences. Revision surgery was performed in 6 of 222 knees (2.7%), with no difference between groups (*p* = 0.44). In the overall cohort, the 15-year Kaplan–Meier survivorship free from revision for all causes was 96.2% (95% CI 93.2–99.4). The estimated 15-year aseptic revision-free survivorship was 98.9% (95% CI 96.9–100) in the CR group and 95.6% (95% CI 91.4–100) in the PS group. Survivorship did not differ significantly between the CR and PS groups for either all-cause or aseptic revision (log-rank *p* = 0.99 and *p* = 0.99, respectively). However, only six revision events occurred, limiting the statistical power to exclude a clinically meaningful between-group difference.

**Conclusions:**

Single-radius TKA demonstrated favorable long-term clinical outcomes and high survivorship in an Asian population. No significant between-group differences were identified in clinical outcomes or survivorship between the CR and PS groups.

## Introduction

The single-radius design of total knee arthroplasty (TKA) has been developed to optimize knee function, and prior studies have demonstrated favorable clinical outcomes and long-term survivorship [1–4]. Many mid- to long-term comparative studies and meta-analyses have reported no significant differences in outcomes between cruciate-retaining (CR) and posterior-stabilized (PS) designs [5–8].

In single-radius TKA, both CR and PS designs are widely used [1, 2, 4]. However, it remains unclear whether differences in posterior cruciate ligament (PCL) management result in meaningful differences in long-term outcomes within a single-radius system [6, 10]. Although these designs share the same single-radius femoral geometry, they differ in the mechanism of sagittal stability. In CR TKA, the retained PCL contributes to stability and femoral rollback during flexion, whereas in PS TKA, these functions are primarily provided by the cam-post mechanism [9–11]. Despite these biomechanical differences, previous comparative studies have not consistently demonstrated clinically meaningful differences between CR and PS designs [5, 8]. Long-term comparative evidence specific to single-radius TKA remains limited, particularly for direct comparisons of CR and PS designs within the same implant platform [5]. Moreover, to our knowledge, comparative data with a minimum follow-up of 10 years are particularly scarce in Asian populations. Because high-flexion activities, including squatting and floor sitting, are common in many Asian patients, these functional demands may influence clinical outcomes and long-term implant durability [12–14]. Therefore, long-term evaluation of CR and PS designs within the same implant platform may help clarify whether differences in PCL management and sagittal stability are associated with clinically meaningful differences in outcomes or implant durability.

Accordingly, the purpose of this study was to evaluate the long-term clinical and radiologic outcomes and survivorship of single-radius TKA in an Asian population and to compare CR and PS designs within the same implant platform with a minimum follow-up of 10 years. We hypothesized that both designs would demonstrate favorable long-term clinical outcomes and survivorship, with no differences between the CR and PS groups in an Asian population.

## Materials and methods

### Patient selection

**Fig. 1:**
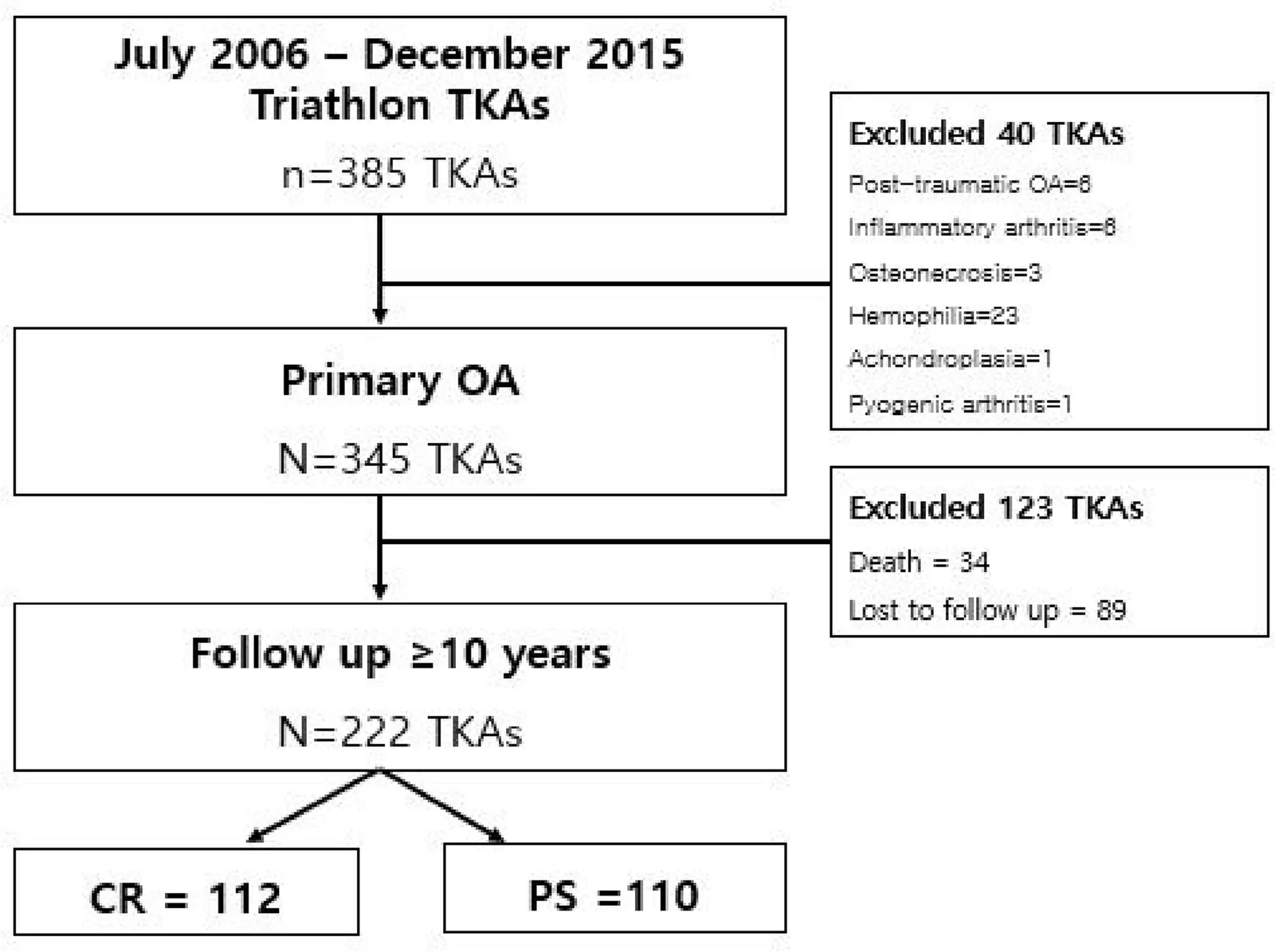
Flowchart of patient selection for the study cohort. A total of 385 total knee arthroplasties (TKAs) performed using a single-radius prosthesis between July 2006 and December 2015 were assessed for eligibility. After exclusion of 40 knees with diagnoses other than primary osteoarthritis and 123 knees because of death or loss to follow-up before 10 years, 222 knees were included in the final analysis. The final cohort comprised 112 cruciate-retaining and 110 posterior-stabilized TKAs. OA, osteoarthritis; TKA, total knee arthroplasty; CR, cruciate-retaining; PS, posterior-stabilized

This retrospective study included consecutive patients who underwent primary TKA using a single-radius design at our institution between July 2006 and December 2015. The inclusion criterion was primary TKA performed for primary osteoarthritis using the single-radius implant system. Exclusion criteria included diagnoses other than primary osteoarthritis and death or loss to follow-up before 10 years. After applying the criteria, 222 knees (152 patients) were finally enrolled. Of these, there were 112 knees (50.5%) with the CR design and 110 knees (49.5%) with the PS design (Fig. 1). Baseline demographic and clinical variables were collected through a retrospective review of medical records and preoperative radiographs.

### Surgical procedures

All surgeries were performed by a single senior surgeon who performed more than 200 primary TKAs annually, using the single-radius Triathlon (Stryker, Mahwah, NJ) prosthesis system. All procedures used conventional manual instrumentation and followed mechanical alignment principles. A mini-mid vastus approach was used for all surgeries [15]. A modified gap technique was used for gap and soft-tissue balancing [16]. The PS design was used in patients with a coronal deformity of > 15°, a fixed flexion contracture (FC) of > 20°, or PCL insufficiency [17, 18]. PCL insufficiency was assessed preoperatively by clinical examination, including the posterior drawer test and posterior sag sign. Intraoperative flexion and extension gaps were assessed using a tensioner after completion of bone resection, osteophyte removal, and soft-tissue balancing [16]. When a CR design was initially attempted, conversion to the PS design was performed if residual FC persisted or if intraoperative gap assessment demonstrated that the flexion gap remained at least 2 mm tighter than the extension gap despite adjustment of the posterior tibial slope and graded PCL release [9, 17, 19]. The posterior tibial slope was created using a conventional tibial cutting guide, with target slopes of 5° for CR TKA and 3° for PS TKA [20, 21]. Patellar resurfacing was performed selectively when either Outerbridge grade III or IV patellar cartilage degeneration or preoperative anterior knee pain was present, provided that the patellar thickness was ≥ 20 mm. Patellae with a thickness of < 20 mm were not resurfaced [22]. Postoperative pain management and rehabilitation protocols were identical for all cases.

### Clinical and radiologic evaluations

Clinical and radiologic outcomes were assessed and compared between the CR and PS groups. Clinical evaluations were conducted preoperatively, at 6 weeks, 3 months, 6 months, 1 year, and annually thereafter, and at the final follow-up, using the Knee Society Knee Score (KSKS) and the Knee Society Function Score (KSFS) [23]. The prespecified primary clinical outcome was the KSFS at the final follow-up. All other clinical and radiographic measures were considered secondary outcomes. At the final follow-up, patient-reported outcome measures included the Western Ontario and McMaster Universities Osteoarthritis Index (WOMAC) and the Forgotten Joint Score (FJS) [24, 25]. FC and range of motion (ROM) of the knee were measured in the supine position using a goniometer. Based on the satisfaction-anchor estimates reported by Lee et al., minimal clinically important difference (MCID) thresholds of 5.9 points for the KSKS and 6.4 points for the KSFS were used [26]. A postoperative FJS of ≥ 22 points was used as the patient-acceptable symptom state (PASS) threshold [27]. These thresholds were used to assess clinically meaningful improvement or an acceptable symptom state, and achievement rates were compared between groups.

Radiologic evaluations included long-leg standing radiographs from the pelvis to the ankle joint, as well as weight-bearing anteroposterior and lateral views. The alignment of the lower limb was assessed using the hip–knee–ankle angle (HKAA) [28]. Implant alignment was assessed on standing radiographs using the *α*, *β*, *γ*, and *δ* angles according to the Knee Society radiographic evaluation system [29]. Radiographic measurements were independently performed by two orthopedic surgeons, and interobserver reliability for continuous measurements was assessed using the intraclass correlation coefficient (ICC). Prosthesis loosening was defined by intraoperative confirmation or radiographic evidence of component migration or progressive radiolucent lines at the implant fixation interface [30].

### Complications and revisions

All intraoperative, early, and late postoperative complications were recorded by review of the medical records and operative notes. Complications were classified according to the standardized list and definitions proposed by the Knee Society [30]. Instability was defined as abnormal and excessive displacement of the prosthetic joint resulting in clinical failure and was classified according to the predominant position in which instability occurred, including extension, flexion, mid-flexion, recurvatum, and global instability [31, 32].

Revision surgery was defined as any subsequent operation involving exchange, removal, or addition of one or more prosthetic components, including the femoral component, tibial component, and polyethylene (PE) insert [33]. In cases of revision surgery, the causes of failure and the time to revision were recorded.

### Survivorship

Kaplan–Meier survivorship analysis was performed to generate survivorship curves with 95% confidence intervals (CIs). Time-to-event was calculated from the date of the index primary TKA to the date of revision surgery, or to the date of the final follow-up for censored cases. Implant survivorship at 15 years was evaluated using revision for all causes as the primary endpoint. The secondary endpoint was aseptic revision, defined as revision excluding infection, with aseptic causes analyzed separately [34]. Patients who underwent revision surgery before 10 years of follow-up were not excluded. Instead, they were retained in the cohort and counted as events at the time of revision [35].

### Data analyses

Because the sample size was fixed by the number of eligible cases in this retrospective cohort, an sensitivity power analysis was performed using G*Power (version 3.1.9.7) to assess the statistical power of the available sample for the prespecified primary clinical outcome, KSFS. The effect size (Cohen’s *d* = 0.47) was derived from a previous long-term comparative study of CR and PS TKA [36]. With 112 knees in the CR group and 110 knees in the PS group, the available cohort provided 93.6% power at a two-sided *α* level of 0.05 to detect this effect size. Categorical variables were compared using the Chi-squared test or Fisher’s exact test, as appropriate. For continuous variables, the Student’s *t*-test was employed for normally distributed data, and the Mann-Whitney *U* test was employed for non-normally distributed data. Changes from preoperative to the final follow-up in the overall cohort were assessed using paired *t*-tests for normally distributed variables and Wilcoxon signed-rank tests for non-normally distributed variables as appropriate. To adjust for baseline imbalances between the CR and PS groups, inverse probability of treatment weighting (IPTW) was applied. Propensity scores were estimated using a logistic regression model including age, sex, BMI, HKAA, KSKS, KSFS, FC, maximum flexion, and ROM. Stabilized weights were used and truncated at the 1 st and 99th percentiles to reduce the influence of extreme weights. Covariate balance after weighting was confirmed by standardized mean differences (SMDs) < 0.10 for all covariates. Clinical outcomes were compared using IPTW-weighted regression models with robust standard errors. To account for the potential inflation of the family-wise Type I error rate due to multiple comparisons, a post hoc Holm adjustment was applied simultaneously to the clinical outcomes, including KSFS, KSKS, FC, maximum flexion, ROM, WOMAC, and FJS. Kaplan–Meier survivorship curves were compared using the log-rank test. A *p*-value < 0.05 was considered statistically significant. Statistical analyses were performed using Statistical Package for the Social Sciences (SPSS) software version 23 (IBM Corp, Armonk, NY).

## Results

In the overall cohort, the mean age at surgery was 67.7 ± 6.4 years, and the mean follow-up duration was 13.2 ± 2.8 years. Tables 1 and 2 present baseline demographic characteristics and preoperative clinical characteristics according to prosthesis design. The mean follow-up period was 13.5 ± 2.6 years in the CR group and 12.8 ± 2.9 years in the PS group (*p* = 0.142) (Table 1). Preoperatively, the CR group had a higher KSKS (*p* < 0.001) and greater ROM than the PS group (*p* < 0.001). The HKAA showed greater varus alignment in the PS group than in the CR group (*p* = 0.028). Other baseline characteristics did not differ significantly between groups (Table 2).

**Table 1:** Demographic and operative characteristics

Demographic	CR	PS	*p*-Value
Knees (*n*)	112	110	
Age (years, mean ± SD)	67.1 ± 6.5	68.2 ± 6.2	0.180
Follow-up period (years, mean ± SD)	13.5 ± 2.6	12.8 ± 2.9	0.142
Sex (male: female)	4:108	6:104	0.724
Body mass index (mean ± SD)	28.1 ± 3.4	28.0 ± 3.9	0.839
HKAA (degree, mean ± SD)	−8.1 ± 4.9	−9.4 ± 5.3	0.028^*^
Patellar resurfacing (*n*, %)	81 (72.3%)	82(74.5%)	0.708

Negative values for HKAA indicate varus alignment. *CR* cruciate-retaining, *PS* posterior-stabilized, *HKAA* hip–knee–ankle angle, *SD* standard deviation.

^*^Statistically significant with *p* < 0.05

**Table 2:** Preoperative clinical scores and range of motion

Preoperative clinical scores and ROM	CR		PS		*p*-Value	
KSFS (mean ± SD)		50.8 ± 13.1		47.1 ± 14.9		0.220
KSKS (mean ± SD)		51.9 ± 16.3		43.1 ± 16.7		< 0.001^*^
Flexion contracture (degree, mean ± SD)		5.9 ± 5.7		9.9 ± 8.7		< 0.001^*^
Maximum flexion (degree, mean ± SD)		131.5 ± 9.8		126.1 ± 14.8		0.010^*^
ROM (degree, mean ± SD)		125.7 ± 13.0		116.1 ± 18.7		< 0.001^*^

*CR* cruciate-retaining, *PS* posterior-stabilized, *KSKS* Knee Society knee score, *KSFS* Knee Society Function Score, *ROM* range of motion, *SD* standard deviation.

^*^Statistically significant with *p* < 0.05

**Fig. 2:**
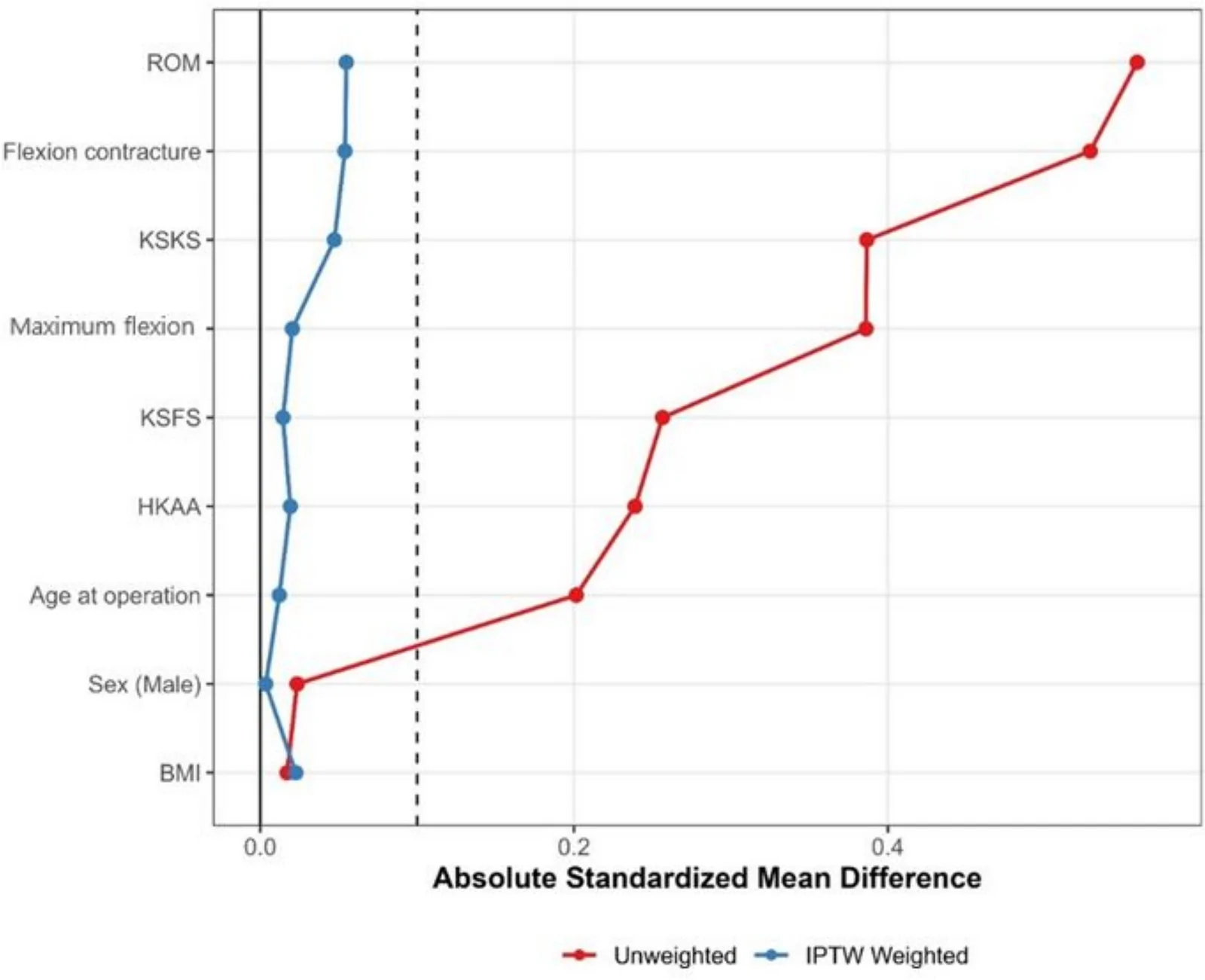
Covariate balance before and after inverse probability of treatment weighting. Absolute standardized mean differences for baseline covariates are presented before and after inverse probability of treatment weighting (IPTW). The vertical dashed line indicates the prespecified threshold of 0.10 for acceptable covariate balance. After weighting, all baseline covariates were below this threshold. *IPTW* inverse probability of treatment weighting, *ROM* range of motion, *KSKS* Knee Society Knee Score, *KSFS* Knee Society Function Score, *HKAA* hip–knee–ankle angle, *BMI* body mass index

Given these baseline imbalances, IPTW was applied. After weighting, covariate balance improved substantially, with SMDs < 0.10 for all covariates (Fig. 2; Table 3).

**Table 3:** Covariate balance before and after inverse probability of treatment weighting

Covariate	Unweighted SMD	IPTW-weighted SMD
ROM	0.56	0.05
Flexion contracture	0.53	0.05
KSKS	0.51	0.05
Maximum flexion	0.39	0.02
KSFS	0.26	0.01
HKAA	0.24	0.02
Age at operation	0.20	0.01
Sex (male)	0.02	0.00
BMI	0.02	0.02

Absolute SMDs are presented for each covariate before and after IPTW. An absolute SMD < 0.10 was considered indicative of acceptable covariate balance. *SMD*, standardized mean difference, *IPTW* inverse probability of treatment weighting, *ROM* range of motion, *KSKS* Knee Society Knee Score, *KSFS* Knee Society Function Score, *HKAA* hip–knee–ankle angle, *BMI* body mass index

The prespecified primary clinical outcome was the KSFS at the final follow-up. After IPTW adjustment, the mean KSFS was 82.1 ± 21.5 in the CR group and 81.1 ± 19.1 in the PS group, with a weighted mean difference of −1.02 points (95% CI −7.24 to 5.21; *p* = 0.747). Thus, no significant between-group difference was detected in the primary clinical outcome.

**Fig. 3:**
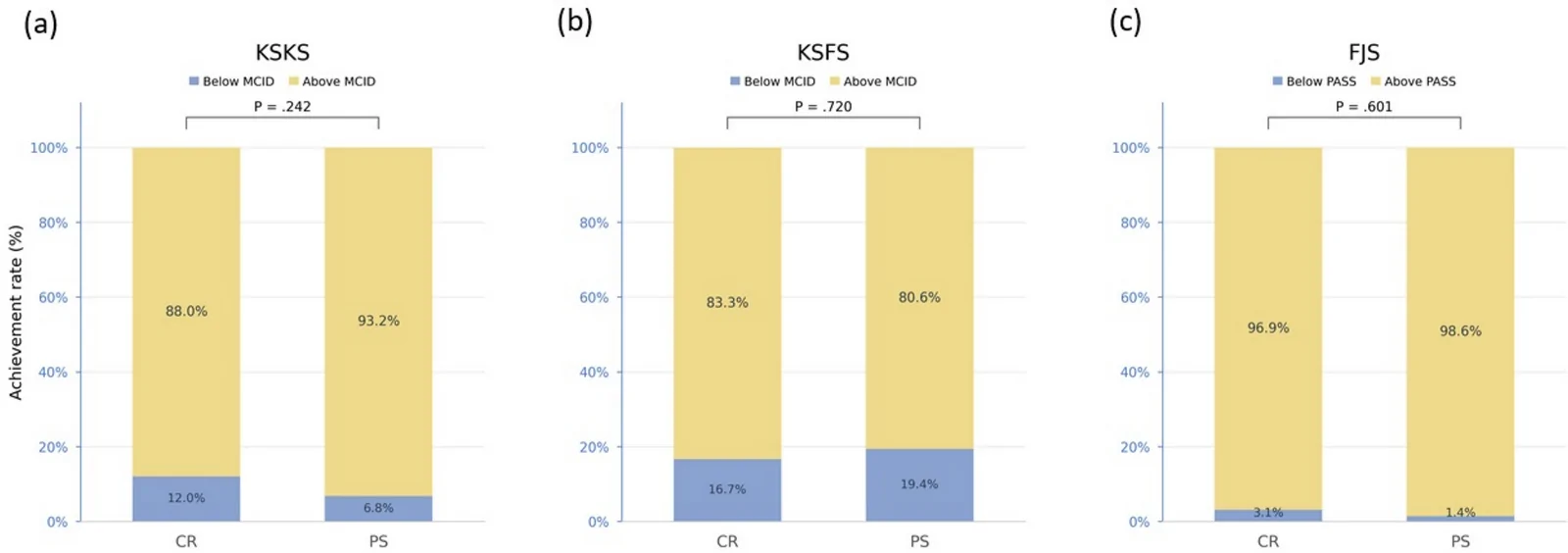
Achievement of clinically meaningful outcomes at final follow-up. Bar plots show the achievement rates of clinically meaningful outcomes in the cruciate-retaining and posterior-stabilized groups: (**a**) minimal clinically important difference (MCID) for the Knee Society Knee Score (KSKS), (**b**) MCID for the Knee Society Function Score (KSFS), and (**c**) patient-acceptable symptom state (PASS) for the Forgotten Joint Score (FJS). No statistically significant between-group differences were identified in any of these outcomes. *CR* cruciate-retaining, *PS* posterior-stabilized, *MCID* minimal clinically important difference, *PASS* patient-acceptable symptom state, *KSKS* Knee Society Knee Score, *KSFS* Knee Society Function Score, *FJS* Forgotten Joint Score

The remaining clinical and radiographic measures were analyzed as secondary outcomes. In the overall cohort, KSKS, KSFS, and ROM improved significantly from the preoperative assessment to the final follow-up (all *p* < 0.001). No significant between-group differences were detected in the secondary clinical outcomes, including KSKS, FC, maximum flexion, ROM, WOMAC, and FJS (Table 4). After Holm adjustment across all clinical outcomes, all adjusted *p*-values were 1.000, and the interpretation of the primary and secondary clinical outcomes remained unchanged. Furthermore, MCID and PASS achievement rates did not differ significantly between groups for any clinical outcome (all *p* > 0.05) (Fig. 3). Radiologic outcomes showed no statistically significant between-group differences in HKAA, *α* angle, *β* angle, or *γ* angle (all *p* > 0.05). The posterior tibial slope (*δ* angle) was greater in the CR group (4.5 ± 2.2°) than in the PS group (3.3 ± 1.6°) (*p* < 0.001), consistent with the predefined surgical targets of 5° for CR and 3° for PS knees. The pattern of radiologic findings was unchanged after IPTW adjustment (Table 4). The ICC values ranged from 0.88 to 0.94, indicating good-to-excellent interobserver reliability.

**Table 4:** Comparison of clinical and radiologic outcomes between CR and PS designs: Unweighted and IPTW-weighted comparisons

	Unweighted				IPTW-weighted				Holm-adjusted
Outcomes	CR	PS	Mean difference (PS − CR, 95% CI)	*p*-Value	CR	PS	Mean difference (PS − CR, 95% CI)	*p*-Value	*p*-Value
Clinical outcomes									
FC (degree)	−0.9 ± 2.2	−0.6 ± 1.8	0.25 (−0.29, 0.79)	0.299	−0.8 ± 2.3	−0.9 ± 2.1	−0.16 (−0.89, 0.58)	0.676	1.000
Maximum flexion (degree)	137.3 ± 8.7	137.1 ± 8.4	−0.24 (−2.53, 2.05)	0.885	137.0 ± 9.0	137.7 ± 8.1	0.65 (−1.89, 3.18)	0.615	1.000
ROM (degree)	138.2 ± 9.7	137.7 ± 9.0	−0.49 (−2.99, 2.02)	0.924	137.8 ± 10.1	138.6 ± 9.0	0.80 (−2.09, 3.70)	0.586	1.000
KSFS	82.7 ± 20.2	79.4 ± 20.5	−3.34 (−8.79, 2.11)	0.162	82.1 ± 21.5	81.1 ± 19.1	−1.02 (−7.24, 5.21)	0.747	1.000
KSKS	83.5 ± 13.9	80.5 ± 15.0	−3.01 (−6.90, 0.87)	0.125	84.2 ± 13.8	81.3 ± 14.8	−2.92 (−7.17, 1.33)	0.176	1.000
WOMAC	32.4 ± 30.9	31.7 ± 27.0	−0.71 (−10.29, 8.86)	0.510	33.8 ± 31.6	28.7 ± 25.5	−5.15 (−15.91, 5.61)	0.346	1.000
FJS	78.4 ± 18.8	78.9 ± 15.7	0.50 (−5.43, 6.42)	0.954	78.2 ± 18.6	79.5 ± 13.8	1.29 (−4.68, 7.26)	0.670	1.000
Radiologic outcomes									
HKAA (degree)	−0.2 ± 2.6	−0.7 ± 2.5	−0.50 (−1.18, 0.18)	0.117	−0.4 ± 2.8	−0.5 ± 2.4	−0.09 (−0.90, 0.71)	0.819	
*α* angle (degree)	95.8 ± 1.8	96.0 ± 2.1	0.23 (−0.40, 0.87)	0.467	95.6 ± 1.8	96.0 ± 2.0	0.42 (−0.25, 1.08)	0.220	
*β* angle (degree)	88.9 ± 1.3	88.8 ± 1.9	−0.06 (−0.60, 0.49)	0.306	89.0 ± 1.4	88.9 ± 2.0	−0.07 (−0.72, 0.58)	0.829	
*γ* angle (degree)	2.9 ± 2.1	3.3 ± 2.4	0.39 (−0.30, 1.09)	0.175	2.8 ± 2.2	3.2 ± 2.4	0.36 (−0.42, 1.13)	0.366	
*δ * angle (degree)	4.5 ± 2.2	3.3 ± 1.6	−1.23 (−1.74, −0.72)	< 0.001^*^	4.4 ± 2.1	3.3 ± 1.6	−1.05 (−1.60, −0.50)	< 0.001^*^	

Data are presented as mean ± standard deviation unless otherwise indicated. Mean differences were calculated as PS − CR and are presented with 95% confidence intervals. After-IPTW estimates were obtained using inverse probability of treatment weighting. Negative HKAA values indicate varus alignment. *CR* cruciate-retaining, *PS* posterior-stabilized, *IPTW* inverse probability of treatment weighting, *FC* flexion contracture, *ROM* range of motion, *KSKS* Knee Society Knee Score, *KSFS* Knee Society Function Score, *WOMAC* Western Ontario and McMaster Universities Osteoarthritis Index, *FJS* Forgotten Joint Score, *HKAA* hip–knee–ankle angle, *CI* confidence interval, *SD* standard deviation.

^*^Statistically significant at p < 0.05

Table 5 presents overall complications. Complications occurred in 5 of 112 knees (4.5%) in the CR group and 10 of 110 knees (9.1%) in the PS group, with no statistically significant between-group difference (*p* = 0.191). Revision surgery was performed in 6 of 222 knees (2.7%). Revision was required in two knees (1.8%) in the CR group and four knees (3.6%) in the PS group, with no statistically significant between-group difference (*p* = 0.44). Both instability cases demonstrated clinically evident laxity in extension and were classified as extension instability. The diagnosis was confirmed intraoperatively at the time of revision, and both knees were treated with PE insert exchange. Aseptic revision cases are presented in Table 6.

**Table 5:** Complications

Complications	Number
Aseptic tibial loosening	3 (3 PS)
Instability	2 (1 CR, 1 PS)
Stiffness (required MUA)	4 (2 CR, 2 PS)
PJI	1 (1 CR)
Periprosthetic fracture Distal femur Patella	5 (1 CR, 4 PS) 3 (1 CR, 2 PS) 2 (2 PS)

*CR* cruciate-retaining, *PS* posterior-stabilized, *PJI* periprosthetic joint infection, *MUA *manipulation under anesthesia

**Table 6:** Aseptic revision cases after single-radius total knee arthroplasty

Design	Time to revision (year)	Reason for revision	Surgical procedure
PS	4.9	Aseptic tibial loosening	Tibial component revision
PS	6.1	Aseptic tibial loosening	Tibial component revision
PS	10.7	Aseptic tibial loosening	Tibial component revision
PS	10.1	Instability	Polyethylene change
CR	10.1	Instability	Polyethylene change

*CR* cruciate-retaining, *PS* posterior-stabilized

**Fig. 4:**
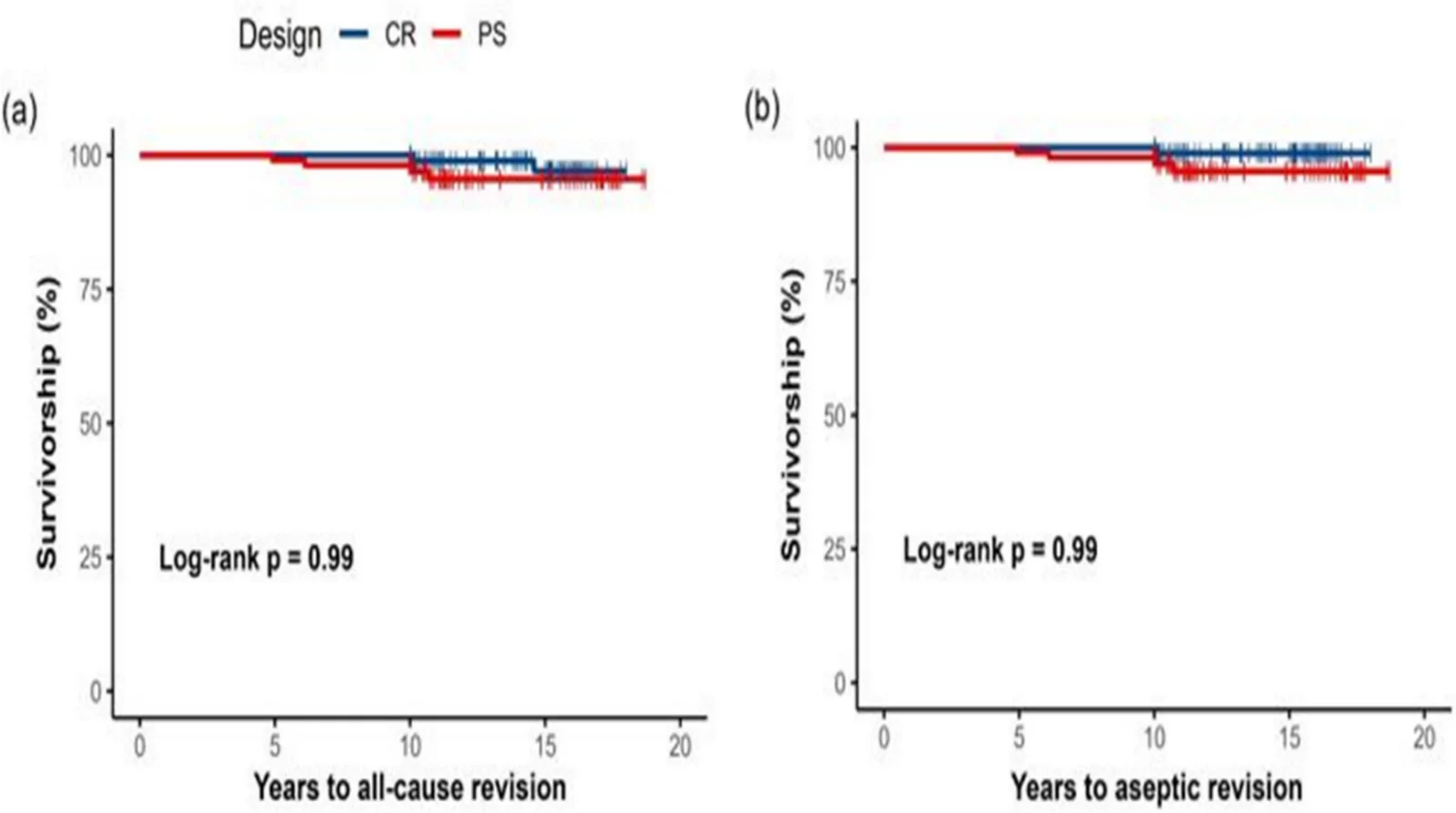
Kaplan–Meier survivorship curves comparing cruciate-retaining and posterior-stabilized designs. Kaplan–Meier curves compare revision-free survivorship between the cruciate-retaining and posterior-stabilized designs using (**a**) revision for all causes and (**b**) aseptic revision as endpoints. No statistically significant between-group differences were observed for revision for all causes (log-rank *p* = 0.99) or aseptic revision (log-rank *p* = 0.99). *CR* cruciate-retaining, *PS* posterior-stabilized

**Fig. 5:**
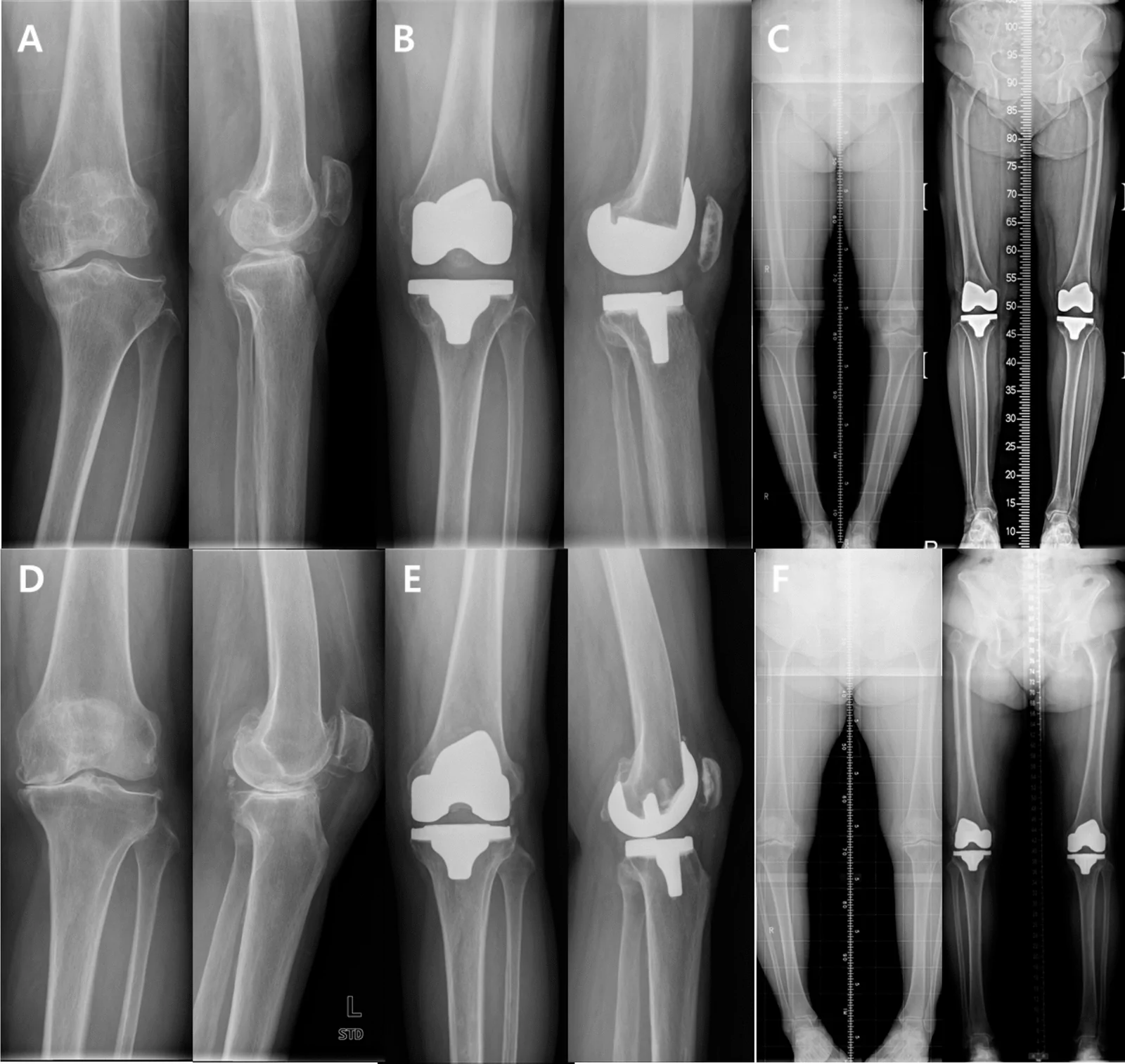
Representative serial radiographs of posterior-stabilized and cruciate-retaining single-radius total knee arthroplasty with the longest follow-up in each group. (**a**) Preoperative anteroposterior and lateral radiographs of the posterior-stabilized (PS) case. (**b**) Postoperative anteroposterior and lateral radiographs after PS total knee arthroplasty. (**c**) Preoperative and final follow-up standing long-leg radiographs of the PS case at 19 years after surgery. (**d**) Preoperative anteroposterior and lateral radiographs of the cruciate-retaining (CR) case. (**e**) Immediate postoperative anteroposterior and lateral radiographs after CR total knee arthroplasty. (**f**) Preoperative and final follow-up standing long-leg radiographs of the CR case at 18 years after surgery. *PS* posterior-stabilized, *CR* cruciate-retaining, *TKA* total knee arthroplasty

In the overall cohort, the 15-year Kaplan–Meier all-cause revision-free survivorship was 96.2% (95% CI 93.2–99.4). The estimated 15-year all-cause revision-free survivorship was 97.0% (95% CI 92.9–100) in the CR group and 95.6% (95% CI 91.4–100) in the PS group. The estimated 15-year aseptic revision-free survivorship was 98.9% (95% CI 96.9–100) in the CR group and 95.6% (95% CI 91.4–100) in the PS group. Kaplan–Meier survivorship analysis showed no statistically significant between-group differences at 15 years for all-cause revision or aseptic revision (Fig. 4). Representative serial radiographs of the PS and CR cases with the longest follow-up are shown in Fig. 5, with follow-up durations of 19 and 18 years, respectively.

## Discussion

This study evaluated the long-term outcomes of single-radius TKA and compared CR and PS designs in an Asian population with a minimum follow-up of 10 years. The principal findings were that single-radius TKA demonstrated favorable long-term clinical outcomes and high implant survivorship after single-radius TKA and that, after adjustment for measured baseline covariates, no statistically significant differences in clinical outcomes or survivorship were detected between the CR and PS groups. Given the limited long-term comparative data on single-radius designs, particularly in Asian populations, this study provides clinically relevant long-term evidence on implant durability and PCL management.

Clinical series have reported favorable clinical outcomes, and long-term follow-up studies have demonstrated excellent implant survivorship exceeding 95% at 10 years [1–3]. These findings support the theoretical advantages of a constant flexion–extension axis and improved extensor efficiency [37, 38]. However most long-term studies of the single-radius design have focused on Western populations [3, 4]. Therefore, the generalizability of these findings to Asian populations remains uncertain [12, 14]. Differences in lifestyle, including frequent high-flexion activities, such as squatting and floor sitting, as well as anatomical characteristics, such as a higher prevalence of constitutional varus alignment, may influence joint kinematics, implant loading, and long-term durability [12, 14]. Moreover, long-term data on single-radius TKA in Asian populations remain limited. This study extends the evidence in an Asian population by evaluating both CR and PS designs in single-radius TKA and by demonstrating favorable long-term outcomes and high 15-year revision-free survivorship within the range reported in Western cohorts.

Additionally, this study compared CR and PS designs within the same single-radius system under real-world clinical indications [39, 40]. Comparing the two designs within the same implant platform reduces heterogeneity related to differences in implant geometry and system-specific characteristics. In contrast to randomized or restricted comparative studies that generally enroll patients with an intact PCL and limited deformity who are eligible for either design, this study included a broader, indication-driven cohort that reflects routine clinical practice [5, 7, 36]. In our cohort, implant selection was based on preoperative and intraoperative findings, with PS designs preferentially used in knees with more severe deformity, FC, PCL insufficiency, or persistent intraoperative flexion–extension gap imbalance when a CR design was initially attempted [10, 17, 41]. Consequently, the PS group had worse baseline clinical and radiologic characteristics than the CR group. After IPTW adjustment, no significant differences in long-term clinical outcomes were detected between the groups. The greater posterior tibial slope observed in the CR group reflected the predefined surgical targets of 5° for CR and 3° for PS knees rather than an independent radiographic effect of implant design. This difference is consistent with design-specific gap-balancing principles. A greater posterior tibial slope in CR TKA may reduce PCL tension and help avoid an excessively tight flexion gap, whereas a smaller posterior slope in PS TKA may help prevent excessive flexion-gap laxity and subsequent flexion instability [19, 21]. No measurable clinical or survivorship consequence of this difference was identified. However, IPTW balanced only the measured baseline covariates and could not account for unmeasured indication-related factors, including intraoperative PCL quality, soft-tissue condition, and real-time flexion–extension gap balance. Residual indication bias and unmeasured confounding therefore remain, and the adjusted findings should not be interpreted as evidence of equivalence, noninferiority, interchangeability, or a causal effect of implant design.

Recent long-term studies and meta-analyses have shown no consistent pattern regarding survivorship differences between CR and PS designs, although some registry-based analyses have suggested a higher risk of revision in PS designs, particularly due to aseptic loosening [5, 6, 42]. From a mechanistic perspective, revision for any cause may not fully reflect differences attributable to implant design, as certain failure modes, such as periprosthetic joint infection or traumatic periprosthetic fracture, are not necessarily attributable to implant design [30, 43, 44]. In this context, aseptic implant-related failure may be a more relevant endpoint for comparing CR and PS designs [42, 45]. In the current study, no statistically significant between-group difference was detected in aseptic revision-free survivorship. However, all three cases of aseptic tibial loosening occurred in the PS group, whereas no aseptic loosening occurred in the CR group. This pattern is clinically noteworthy but should be interpreted cautiously because of the small number of events and the indication-driven differences between the groups. In PS TKA, cam-post engagement provides posterior stability and guides femoral translation during flexion, but it may also increase anteroposterior constraint, torsional loading, and stress transfer to the tibial component-bone interface [11]. Cam-post loading, particularly during high-flexion activities, may theoretically contribute to micromotion and increased stress at the tibial fixation interface [46, 47]. However, because the numbers of all-cause and aseptic revision events were small, this study had limited statistical power to detect between-group differences or to exclude a clinically meaningful survivorship difference. Therefore, the high log-rank *p*-values should be interpreted as statistically inconclusive rather than as evidence of equivalent survivorship. Moreover, the PS group included knees with more severe preoperative deformity, greater FC, and potentially more complex soft-tissue imbalance, which may independently increase surgical complexity or implant-interface loading [48, 49]. Therefore, implant design cannot be isolated as the cause of loosening, and the exclusive occurrence of aseptic tibial loosening in the PS group should be considered hypothesis-generating rather than evidence of a causal design-related difference.

From a surgical perspective, CR TKA is generally considered more technically demanding than PS TKA because it requires PCL preservation while achieving appropriate gap balance and soft-tissue tension [10, 20, 21]. For this reason, PS designs remain widely used in clinical practice to achieve more predictable stability and technical reproducibility, particularly in severely deformed knees [10, 41]. Although the PS group had more severe preoperative disease, no statistically significant disadvantage in long-term clinical outcomes was detected after adjustment for measured covariates [10, 41]. Recent advances in TKA have shifted the focus from conventional mechanical alignment toward more patient-specific approaches, including kinematic alignment, functional alignment, and robotic-assisted techniques [50–52]. These contemporary concepts aim to better restore native knee kinematics by preserving individual anatomy and soft-tissue balance [50–52]. In this context, CR designs have gained renewed interest because PCL preservation, more native knee kinematics, and less bone resection may be more consistent with these principles [11, 50]. These findings suggest that favorable long-term outcomes may be achieved with CR TKA in appropriately selected patients. Accordingly, these long-term findings may provide a useful reference for interpreting future evidence as CR techniques continue to evolve and long-term successful outcomes of kinematically aligned CR TKA become available. Although CR TKA may be conceptually aligned with evolving trends toward more physiologic reconstruction, both CR and PS designs remain clinically appropriate options when selected according to patient-specific indications. Therefore, implant selection should be individualized according to patient characteristics, deformity severity, and the surgeon’s philosophy and proficiency [5, 6, 8].

This study has several limitations. First, this retrospective, nonrandomized study used clinical indications to guide implant selection. Although IPTW achieved balance in measured baseline covariates, residual confounding related to unmeasured or incompletely recorded factors cannot be excluded. Second, only six revision events occurred, including five aseptic revisions. Consequently, the survivorship analyses had limited statistical power, and the absence of statistically significant between-group differences should not be interpreted as evidence of equivalent survivorship. A clinically meaningful difference between CR and PS designs therefore cannot be excluded. Third, multiple secondary clinical and radiographic outcomes were examined without formal multiplicity adjustment. Therefore, the possibility of inflated Type I error cannot be excluded, and the secondary findings should be interpreted as exploratory. Fourth, the predominance of women, the single-center setting, and the use of a specific implant system may limit generalizability. Although the predominance of women is common in Asian populations, differences in lifestyle and skeletal structure may limit the generalizability of these findings to Western populations. In addition, the relatively long enrollment period may have introduced temporal heterogeneity, although all procedures were performed by a single surgeon using a consistent implant system and surgical strategy.

## Conclusions

Single-radius total knee arthroplasty demonstrated favorable long-term clinical outcomes and high survivorship in an Asian population. No statistically significant differences clinical outcomes or survivorship were detected between the CR and PS groups. However, the small number of revision events limited the statistical power to exclude a clinically meaningful difference between designs. These findings suggest that favorable long-term outcomes may be achieved with either CR or PS designs when implant selection is appropriately individualized according to patient-specific indications.

## Data Availability

The datasets used and/or analyzed during the current study are not publicly available, because they contain potentially identifiable clinical information, but are available from the corresponding author on reasonable request and subject to approval by the relevant institutional review board, where applicable.

## Abbreviations

TKA: Total knee arthroplasty
CR: Cruciate-retaining
PS: Posterior-stabilized
PCL: Posterior cruciate ligament
KSKS: Knee Society Knee Score
KSFS: Knee Society Function Score
WOMAC: Western Ontario and McMaster Universities Osteoarthritis Index
FJS: Forgotten Joint Score
ROM: Range of motion
HKAA: Hip–knee–ankle angle
FC: Flexion contracture
MCID: Minimal clinically important difference
PASS: Patient-acceptable symptom state
IPTW: Inverse probability of treatment weighting
SMD: Standardized mean difference
CI: Confidence interval
PE: Polyethylene
PJI: Periprosthetic joint infection
MUA: Manipulation under anesthesia
